# Identification of shared risk loci and pathways for bipolar disorder and schizophrenia

**DOI:** 10.1371/journal.pone.0171595

**Published:** 2017-02-06

**Authors:** Andreas J. Forstner, Julian Hecker, Andrea Hofmann, Anna Maaser, Céline S. Reinbold, Thomas W. Mühleisen, Markus Leber, Jana Strohmaier, Franziska Degenhardt, Jens Treutlein, Manuel Mattheisen, Johannes Schumacher, Fabian Streit, Sandra Meier, Stefan Herms, Per Hoffmann, André Lacour, Stephanie H. Witt, Andreas Reif, Bertram Müller-Myhsok, Susanne Lucae, Wolfgang Maier, Markus Schwarz, Helmut Vedder, Jutta Kammerer-Ciernioch, Andrea Pfennig, Michael Bauer, Martin Hautzinger, Susanne Moebus, Lorena M. Schenk, Sascha B. Fischer, Sugirthan Sivalingam, Piotr M. Czerski, Joanna Hauser, Jolanta Lissowska, Neonila Szeszenia-Dabrowska, Paul Brennan, James D. McKay, Adam Wright, Philip B. Mitchell, Janice M. Fullerton, Peter R. Schofield, Grant W. Montgomery, Sarah E. Medland, Scott D. Gordon, Nicholas G. Martin, Valery Krasnov, Alexander Chuchalin, Gulja Babadjanova, Galina Pantelejeva, Lilia I. Abramova, Alexander S. Tiganov, Alexey Polonikov, Elza Khusnutdinova, Martin Alda, Cristiana Cruceanu, Guy A. Rouleau, Gustavo Turecki, Catherine Laprise, Fabio Rivas, Fermin Mayoral, Manolis Kogevinas, Maria Grigoroiu-Serbanescu, Tim Becker, Thomas G. Schulze, Marcella Rietschel, Sven Cichon, Heide Fier, Markus M. Nöthen

**Affiliations:** 1 Institute of Human Genetics, University of Bonn, Bonn, Germany; 2 Department of Genomics, Life & Brain Center, University of Bonn, Bonn, Germany; 3 Department of Psychiatry (UPK), University of Basel, Basel, Switzerland; 4 Human Genomics Research Group, Department of Biomedicine, University of Basel, Basel, Switzerland; 5 Institute of Medical Genetics and Pathology, University Hospital Basel, Basel, Switzerland; 6 Institute for Genomics Mathematics, University of Bonn, Bonn, Germany; 7 Institute of Medical Microbiology, Immunology and Parasitology, University of Bonn, Bonn, Germany; 8 Institute of Neuroscience and Medicine (INM-1), Research Center Juelich, Juelich, Germany; 9 Department of Psychiatry & Psychotherapy, University of Cologne, Cologne, Germany; 10 Department of Genetic Epidemiology in Psychiatry, Central Institute of Mental Health, Medical Faculty Mannheim/University of Heidelberg, Mannheim, Germany; 11 Department of Biomedicine and Centre for integrative Sequencing, iSEQ, Aarhus University, Aarhus, Denmark; 12 The Lundbeck Foundation Initiative for integrative Psychiatric Research, iPSYCH, Aarhus and Copenhagen, Denmark; 13 National Centre Register-Based Research, Aarhus University, Aarhus, Denmark; 14 German Center for Neurodegenerative Diseases (DZNE), Bonn, Germany; 15 Department of Psychiatry, Psychosomatic Medicine and Psychotherapy, University Hospital Frankfurt am Main, Frankfurt am Main, Germany; 16 Max Planck Institute of Psychiatry, Munich, Germany; 17 Munich Cluster for Systems Neurology (SyNergy), Munich, Germany; 18 University of Liverpool, Institute of Translational Medicine, Liverpool, United Kingdom; 19 Department of Psychiatry, University of Bonn, Bonn, Germany; 20 Psychiatric Center Nordbaden, Wiesloch, Germany; 21 Center of Psychiatry Weinsberg, Weinsberg, Germany; 22 Department of Psychiatry and Psychotherapy, University Hospital Carl Gustav Carus, TU Dresden, Dresden, Germany; 23 Department of Psychology, Clinical Psychology and Psychotherapy, Eberhard Karls University Tübingen, Tübingen, Germany; 24 Institute of Medical Informatics, Biometry and Epidemiology, University Duisburg-Essen, Essen, Germany; 25 Laboratory of Psychiatric Genetics, Department of Psychiatry, Poznan University of Medical Sciences, Poznan, Poland; 26 Department of Cancer Epidemiology and Prevention, Maria Sklodowska-Curie Memorial Cancer Centre and Institute of Oncology Warsaw, Warsaw, Poland; 27 Department of Epidemiology, Nofer Institute of Occupational Medicine, Lodz, Poland; 28 Genetic Epidemiology Group, International Agency for Research on Cancer (IARC), Lyon, France; 29 Genetic Cancer Susceptibility Group, International Agency for Research on Cancer (IARC), Lyon, France; 30 School of Psychiatry, University of New South Wales, Randwick, Australia; 31 Black Dog Institute, Prince of Wales Hospital, Randwick, Australia; 32 Neuroscience Research Australia, Sydney, Australia; 33 School of Medical Sciences Faculty of Medicine, University of New South Wales, Sydney, Australia; 34 Queensland Institute of Medical Research (QIMR), Brisbane, Australia; 35 Moscow Research Institute of Psychiatry, Moscow, Russian Federation; 36 Institute of Pulmonology, Russian State Medical University, Moscow, Russian Federation; 37 Russian Academy of Medical Sciences, Mental Health Research Center, Moscow, Russian Federation; 38 Department of Biology, Medical Genetics and Ecology, Kursk State Medical University, Kursk, Russian Federation; 39 Research Institute for Genetic and Molecular Epidemiology, Kursk State Medical University, Kursk, Russian Federation; 40 Institute of Biochemistry and Genetics, Ufa Scientific Center of Russian Academy of Sciences, Ufa, Russian Federation; 41 Department of Genetics and Fundamental Medicine of Bashkir State University, Ufa, Russian Federation; 42 Department of Psychiatry, Dalhousie University, Halifax, Canada; 43 National Institute of Mental Health, Klecany, Czech Republic; 44 Montreal Neurological Institute, McGill University, Montreal, Canada; 45 Department of Human Genetics, McGill University, Montreal, Canada; 46 McGill Group for Suicide Studies & Douglas Research Institute, Montreal, Canada; 47 Department of Psychiatry, McGill University, Montreal, Canada; 48 Département des sciences fondamentales, Université du Québec à Chicoutimi (UQAC), Saguenay, Canada; 49 Department of Psychiatry, Hospital Regional Universitario, Biomedical Institute of Malaga, Malaga, Spain; 50 Center for Research in Environmental Epidemiology (CREAL), Barcelona, Spain; 51 Biometric Psychiatric Genetics Research Unit, Alexandru Obregia Clinical Psychiatric Hospital, Bucharest, Romania; 52 Institute for Medical Biometry, Informatics and Epidemiology, University of Bonn, Bonn, Germany; 53 Institute of Psychiatric Phenomics and Genomics, Ludwig-Maximilians-University Munich, Munich, Germany; University of Texas Health Science Center at Houston, UNITED STATES

## Abstract

Bipolar disorder (BD) is a highly heritable neuropsychiatric disease characterized by recurrent episodes of mania and depression. BD shows substantial clinical and genetic overlap with other psychiatric disorders, in particular schizophrenia (SCZ). The genes underlying this etiological overlap remain largely unknown. A recent SCZ genome wide association study (GWAS) by the Psychiatric Genomics Consortium identified 128 independent genome-wide significant single nucleotide polymorphisms (SNPs). The present study investigated whether these SCZ-associated SNPs also contribute to BD development through the performance of association testing in a large BD GWAS dataset (9747 patients, 14278 controls). After re-imputation and correction for sample overlap, 22 of 107 investigated SCZ SNPs showed nominal association with BD. The number of shared SCZ-BD SNPs was significantly higher than expected (*p = 1*.*46x10*^*-8*^). This provides further evidence that SCZ-associated loci contribute to the development of BD. Two SNPs remained significant after Bonferroni correction. The most strongly associated SNP was located near *TRANK1*, which is a reported genome-wide significant risk gene for BD. Pathway analyses for all shared SCZ-BD SNPs revealed 25 nominally enriched gene-sets, which showed partial overlap in terms of the underlying genes. The enriched gene-sets included calcium- and glutamate signaling, neuropathic pain signaling in dorsal horn neurons, and calmodulin binding. The present data provide further insights into shared risk loci and disease-associated pathways for BD and SCZ. This may suggest new research directions for the treatment and prevention of these two major psychiatric disorders.

## Introduction

Bipolar disorder (BD) is a severe neuropsychiatric disease characterized by recurrent episodes of mania and depression. BD has an estimated lifetime prevalence of around 1% [1], and a heritability of around 70% [2]. BD shows substantial clinical and genetic overlap with other psychiatric disorders [3, 4]. An analysis of the genome-wide genotype data of the Psychiatric Genomics Consortium (PGC) revealed a 68% genetic correlation between BD and schizophrenia (SCZ), which was the highest correlation with BD of all psychiatric diseases investigated [3]. However, the genes involved in this etiological overlap remain largely unknown.

Although research into BD and SCZ has identified a number of susceptibility genes, the respective biological pathways still await identification. For BD, recent genome wide association studies (GWAS) have identified a number of risk loci [5–13].

For SCZ, a PGC meta-analysis of data from >36,000 patients and 113,000 controls identified 128 independent genome-wide significant single nucleotide polymorphisms (SNPs) in 108 genetic loci [14].

The aim of the present study was to investigate whether these 128 SCZ-associated SNPs also contribute to the development of BD. For this purpose, we performed association testing of these SNPs in our large BD GWAS dataset [12]. In addition, we analyzed whether the genome-wide significant BD-associated SNPs identified in our BD GWAS [12] show association with SCZ.

## Materials and methods

### Sample description

The analyses were performed using data from our previous GWAS of BD (9,747 patients and 14,278 controls) [12]. This GWAS dataset combined: (i) the MooDS data (collected from Canada, Australia, and four European countries); and (ii) the GWAS results for BD of the large multinational PGC [5]. The patients were assigned the following diagnoses (DSM-IV, DSM-IIR, Research Diagnostic Criteria): BD type 1 (n = 8,001; 82.1%); BD type 2 (n = 1,212; 12.4%); schizoaffective disorder (bipolar type; n = 269; 2.8%); and BD not otherwise specified (n = 265, 2.7%) [12]. The study was approved by the local ethics committees of the participating centers (University Hospital Würzburg, Germany; Central Institute of Mental Health, Mannheim, Germany; University of Essen, Germany; Ludwig Maximilians University, Munich, Germany; Prince of Wales Hospital, Sydney, Australia; Queensland Institute of Medical Research, Brisbane, Australia; Poznan University of Medical Sciences, Poland; University of Szczecin, Poland; speciality mood disorders clinics in Halifax and Ottawa, Canada; Russian State Medical University, Moscow, Russian Federation; Kursk State Medical University, Russian Federation; Regional University Hospital of Malaga, Spain; and Instituto Municipal de Asistencia Sanitaria, IMAS-IMIM, Barcelona, Spain) [12]. Written informed consent was obtained from all participants prior to inclusion [12].

### Genome-wide significant loci for SCZ and BD

For the 128 linkage disequilibrium (LD)-independent genome-wide significant SNPs for SCZ, genetic information was obtained from the supplementary information of the SCZ GWAS of the PGC [14]. This is the largest GWAS of SCZ to date.

Genome-wide significant SNPs for BD were obtained from our BD GWAS [12].

### Imputation and meta-analysis

Different reference panels were used for the imputation of the MooDS and PGC BD genotype data (1,000 Genomes Project, February 2012 release; and HapMap phase 2 CEU, respectively). Therefore, the summary statistics of the PGC BD GWAS [5] were imputed using the 1,000 Genomes Project reference panel and ImpG-Summary. The latter is a recently proposed method for the rapid and accurate imputation of summary statistics [15]. This resulted in z-scores for >20 million SNPs. A total of 111 SCZ-associated SNPs could be mapped to the re-imputed PGC BD GWAS data. The remaining variants were either located on the X-chromosome (n = 3), or represented insertions or deletions (n = 14) which could not be imputed by the applied method. In total, 107 of the 111 SCZ-associated SNPs could be identified in the MooDS BD GWAS.

A meta-analysis for these 107 SNPs was then performed by combining the PGC BD GWAS and the MooDS BD GWAS, and using the sample size based strategy implemented in METAL [16].

### Analysis of shared BD-SCZ SNPs

The risk alleles for all nominally significant SNPs in our BD GWAS [12] were compared to those reported in the PGC SCZ GWAS.

The SCZ discovery meta-analysis comprised data from 35,476 patients and 46,839 controls. Our BD GWAS comprised data from 9,747 patients and 14,278 controls [12]. To correct for an overlap between the two studies of around 500 patients and 9,200 controls [17, 18], we applied the framework of a bivariate normal distribution for the z-scores from both studies, corresponding to a specific SNP. Since the significant hits from a study were selected from different chromosomal regions, we assumed that the z-scores within a study are independent. According to the LD Score regression method [19], the mean inflation of the test statistics provides an approximation of the variance of the z-scores. By considering the set of SNPs in the HapMap3 reference panel [20], the calculated variance was approximately 1.82 for SCZ and 1.24 for BD. From equation (16) in Bulik-Sullivan et al. [19] (Supplementary Material), the covariance between z-scores was calculated to be 0.1644, under the assumption of no genetic correlation. This yielded a correlation of approximately 0.109. To confirm the validity of these theoretical calculations, we estimated the covariance of z-scores due to sample overlap by applying the LD Score regression software directly to the results of the PGC SCZ GWAS and our BD GWAS. After restriction to the well-imputed SNPs of HapMap3, the software estimated a covariance of 0.1707. This result provides further evidence that the degree of sample overlap was correctly estimated in the present study.

The z-scores for the 107 SCZ-associated SNPs were extracted from the PGC SCZ discovery study. The corresponding z-scores were extracted from our BD GWAS [12]. Using the values above, the mean and the variance of the normal distribution for the BD z-scores were determined, given the z-scores from the PGC SCZ discovery study. After the transformation of the initial z-scores from our BD GWAS, a total of 22 of 107 z-scores for BD had corresponding two-sided association p-values of <5% (Table 1).

**Table 1:** Schizophrenia-associated SNPs with a p-value of <0.05 in our bipolar disorder GWAS data after correction for sample overlap.

SNP	Chr	Position	Alleles	P BD Meta	P_corr_ BD Meta	P PGC SCZ	Nearby Gene/s
rs75968099	3	36858583	T/C	2.03 x 10^−5^	0.0022	1.05 x 10^−13^	*TRANK1*
rs2535627	3	52845105	T/C	4.68 x 10^−5^	0.0052	4.26 x 10^−11^	*ITIH3-ITIH4*
rs6704641	2	200164252	A/G	0.0030	0.3331	8.33 x 10^−9^	*SATB2*
rs140505938	1	150031490	T/C	0.0032	0.3597	4.49 x 10^−10^	*VPS45*
rs7893279	10	18745105	T/G	0.0043	0.4770	1.97 x 10^−12^	*CACNB2*
rs6704768	2	233592501	A/G	0.0063	0.6991	2.32 x 10^−12^	*GIGYF2*
rs12704290	7	86427626	A/G	0.0075	0.8315	3.33 x 10^−10^	*GRM3*
rs211829	7	110048893	T/C	0.0088	0.9778	3.71 x 10^−8^	*-*
rs3735025	7	137074844	T/C	0.0098	>0.9999	3.28 x 10^−9^	*DGKI*
rs324017	12	57487814	A/C	0.0098	>0.9999	2.13 x 10^−8^	*NAB2*
rs2909457	2	162845855	A/G	0.0109	>0.9999	4.62 x 10^−8^	*SLC4A10-DPP4*
rs9922678	16	9946319	A/G	0.0120	>0.9999	1.28 x 10^−8^	*GRIN2A*
rs950169	15	84706461	T/C	0.0181	>0.9999	1.62 x 10^−11^	*ADAMTSL3*
rs55661361	11	124613957	A/G	0.0301	>0.9999	2.8 x 10^−12^	*NRGN*
rs10043984	5	137712121	T/C	0.0307	>0.9999	1.09 x 10^−8^	*KDM3B*
rs1498232	1	30433951	T/C	0.0323	>0.9999	2.86 x 10^−9^	*LOC101929406*
rs6434928	2	198304577	A/G	0.0351	>0.9999	2.06 x 10^−11^	*SF3B1-COQ10B*
rs2007044	12	2344960	A/G	0.0367	>0.9999	3.22 x 10^−18^	*CACNA1C*
rs8044995	16	68189340	A/G	0.0380	>0.9999	1.51 x 10^−8^	*NFATC3*
rs56205728	15	40567237	A/G	0.0387	>0.9999	4.18 x 10^−9^	*PAK6*
rs2693698	14	99719219	A/G	0.0429	>0.9999	4.8 x 10^−9^	*BCL11B*
rs832187	3	63833050	T/C	0.0465	>0.9999	1.43 x 10^−8^	*THOC7*

Single nucleotide polymorphisms (SNPs) are shown according to their p-values in our bipolar disorder (BD) GWAS [12] following correction for sample overlap. Chromosomal positions refer to genome build GRCh37 (hg19). Abbreviations: Chr, chromosome; P BD Meta, p-value in our BD GWAS [12] after correction for sample overlap; P_corr_ BD Meta, p-value in our BD GWAS [12] after correction for sample overlap and Bonferroni correction for multiple testing; P PGC SCZ, p-value in the PGC schizophrenia GWAS [14].

Analogously, the z-scores for the genome-wide significant BD SNPs were extracted from our BD GWAS [12], and the corresponding z-scores were extracted from the PGC SCZ discovery study. Of the five BD-associated lead SNPs in our BD GWAS, one SNP (rs6550435) was in high LD (r^2^ = 0.897, SNAP [21]) with a genome-wide SCZ-associated SNP (rs75968099), and was thus excluded from this additional analysis. For the remaining four SNPs, the transformation was computed in the other direction. After correction for sample overlap, no BD SNP showed association with SCZ.

Bonferroni correction for multiple testing was performed by multiplying the nominal p-values with the number of investigated SNPs (n = 107+4 = 111).

### Pathway analysis

Pathway analysis for all 22 shared SCZ-BD SNPs was performed using Ingenuity Pathway Analysis (IPA; http://www.ingenuity.com/) [22, 23] and INRICH [24].

In IPA, each gene is represented in a global molecular network, which is designed using information from the Ingenuity Pathway Knowledge Base. ‘Networks’ were generated algorithmically, and on the basis of their connectivity in terms of activation, expression, and transcription. Molecular relationships between genes are represented by connecting lines between nodes, as supported by published data stored in the Ingenuity Pathway Knowledge Base and/or PubMed. For the purposes of the present study, the canonical pathway analysis available in IPA was applied. Here, an SNP is mapped to a gene if it falls within the gene-coding region or within the 2 kilobase (kb) upstream/ 0.5 kb downstream range of the gene-coding region. This resulted in the inclusion of 13 genes in the pathway analysis. Significant pathways were filtered in order to achieve a minimum of two genes per set. The significance of the association between the SNP-associated genes mapped by IPA and the canonical pathway was measured using Fisher’s exact test.

INRICH [24] was used as a secondary pathway analysis tool, as it enables examination of enriched association signals of LD-independent genomic intervals. Gene Ontology (GO) gene sets were extracted from the Molecular Signatures Database (MSigDB), version 5.0 (Broad Institute, http://software.broadinstitute.org/gsea/msigdb/index.jsp, downloaded in September 2015). The size of the extracted gene sets ranged from 10 to 200 genes, resulting in 1,268 target sets for testing. The intervals around the 22 SNPs of interest were based on empirical estimates of LD from PLINK (http://pngu.mgh.harvard.edu/purcell/plink/). SNPs were assigned to genes using 50 kb up- and downstream windows. In total, 21 intervals were tested for the 1,268 target sets.

In IPA, correction for multiple testing was performed using the Benjamini Hochberg method. In INRICH, the empirical gene set p-value was corrected for multiple testing using bootstrapping-based re-sampling.

## Results

A total of 107 of the 128 SCZ-associated SNPs could be mapped to both the re-imputed PGC BD GWAS and the MooDS BD GWAS data. A meta-analysis of these 107 SNPs was then performed using METAL [16].

After correction for sample overlap, 22 of the 107 SCZ-associated SNPs showed nominally significant p-values in our BD GWAS (Table 1, S1 Table). For all 22 SNPs, the direction of the effect was identical to that observed in the PGC SCZ GWAS [14]. Of the five genome-wide significant BD-associated SNPs identified in our BD GWAS, one SNP (rs6550435) was in high LD (r^2^ = 0.897) with a genome-wide SCZ-associated SNP (rs75968099). None of the remaining four genome-wide significant BD-associated SNPs showed a nominally significant association with SCZ after correction for sample overlap (data not shown).

The number of SCZ SNPs with a p-value of <0.05 in our BD GWAS (n = 22) was significantly higher than expected (*p = 1*.*46x10*^*-8*^, binomial test). This provides further evidence that SCZ-associated loci contribute to the development of BD.

The most strongly associated SNP was located near the gene *TRANK1* (Table 1, *p = 2*.*03x10*^*-5*^), which is a reported genome-wide significant risk gene for BD [7, 12]. The other nominally associated SCZ-BD SNPs implicated loci which contain interesting candidate genes for BD and SCZ. These include the chromatin remodeling gene *SATB2*, the glutamate receptor genes *GRM3* and *GRIN2A*, and the calcium channel subunit gene *CACNB2*. The latter is a reported genome-wide significant risk gene for a number of psychiatric disorders, including BD and SCZ [17].

After Bonferroni correction for multiple testing, two SNPs (rs75968099, rs2535627) showed significant association with BD (*p*_*corr*_ *= 2*.*25x10*^*-3*^ and *p*_*corr*_ *= 5*.*19x10*^*-3*^, respectively).

**Fig 1:**
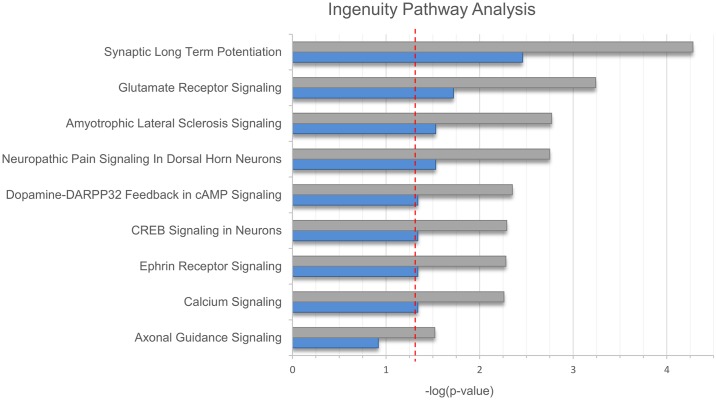
Results of the Ingenuity Pathway Analysis. Results of the Ingenuity Pathway Analysis (IPA) are shown in bar plot format. The x-axis shows negative logarithmic enrichment p-values for all associated pathways containing two and more genes prior to- (gray) and after- (blue) Benjamini Hochberg correction for multiple testing. The red horizontal line indicates a p-value of 0.05.

**Fig 2:**
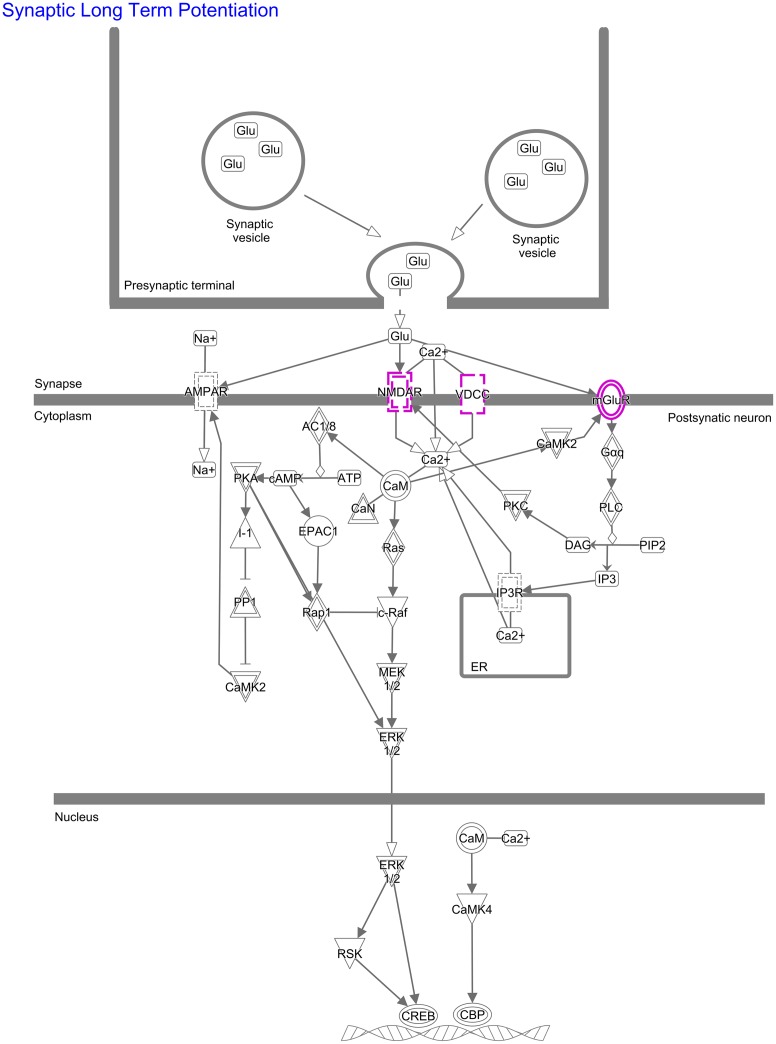
IPA pathway synaptic long term potentiation. Results of the Ingenuity pathway analysis (IPA) for the pathway “Synaptic Long Term Potentiation” are shown. Shared schizophrenia-bipolar disorder associated genes (*GRIN2A*, *GRM3*, *CACNA1C*) are highlighted in purple.

Pathway analysis using IPA revealed nine pathways with nominally significant enrichment (Fig 1). Of these, eight remained significantly enriched after Benjamini Hochberg correction for multiple testing. The pathway with the strongest enrichment was synaptic long term potentiation (*p*_*corr*_ = 0.003, Fig 2, S2 Table). In addition, significant enrichment was found for glutamate receptor- and calcium signaling; neuropathic pain signaling in dorsal horn neurons; and CREB signaling in neurons.

These findings are consistent with previous pathway analyses of BD and SCZ [5, 25–27]. The present analysis also confirmed the glutamatergic signaling pathway, which was considered provisional in a recent review [28].

**Fig 3:**
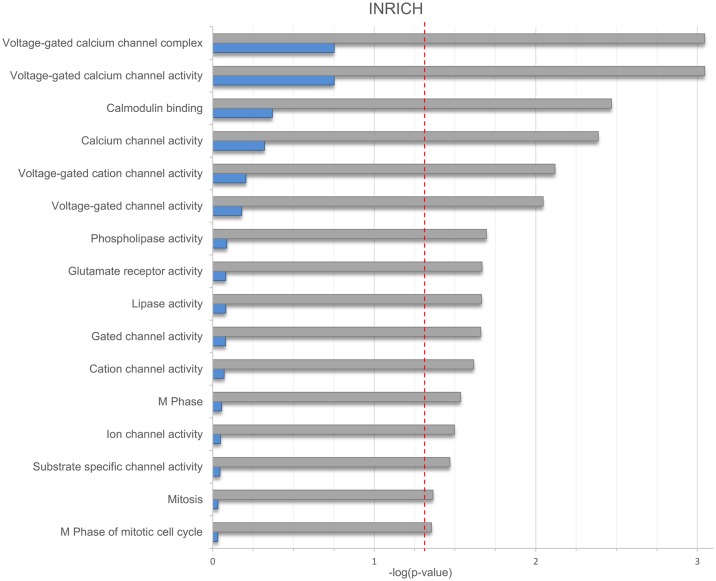
Results of the INRICH pathway analysis. Results of the INRICH pathway analysis are shown in bar plot format. The x-axis shows negative logarithmic enrichment p-values for all nominally associated pathways containing two and more genes prior to- (gray) and after- (blue) correction for multiple testing. The red horizontal line indicates a p-value of 0.05.

Pathway analysis using INRICH identified a total of 16 nominally significant gene-sets, which showed partial overlap in terms of the underlying genes. The enriched gene-sets include voltage-gated calcium channel complex/activity; calmodulin binding; glutamate receptor activity; and M phase of the mitotic cell cycle (Fig 3). None of these gene-sets remained significantly enriched for associations after correction for multiple testing (Fig 3, S3 Table).

## Discussion

The present analyses revealed a significant enrichment of BD-associated SNPs within known SCZ-associated loci (*p = 1*.*46x10*^*-8*^). This is consistent with previous reports of overlapping genetic susceptibility for BD and SCZ [4, 29, 30].

The most strongly associated SNP was located near *TRANK1*, which is a reported genome-wide significant risk gene for BD [7]. The second SNP with significant BD association after correction for multiple testing (rs2535627, Table 1) was located in a genomic region on chromosome 3. This region contains multiple genes, including inter-alpha-trypsin inhibitor heavy chain 3 (*ITIH3*) and -4 (*ITIH4*). Common variation at the *ITIH3*-*ITIH4* region has been identified as a genome-wide significant risk factor for five different psychiatric disorders, including SCZ and BD [17].

Interestingly, the GWAS index SNP rs2535627 represents a Bonferroni-significant fetal brain methylation quantitative trait locus (mQTL), as it has been associated with DNA methylation at cg11645453. The latter is located in the 5’ untranslated region of *ITIH4* [31]. This suggests that the SCZ-BD associated SNP rs2535627 might contribute to disease susceptibility by altering the expression of ITIH4 in the brain [32]. This hypothesis is supported by a recent study, which found that the G-allele of the SNP rs4687657—which is in moderate LD with rs2535627 (r^2^ = 0.426, D’ = 1.000, SNAP [21])—was significantly associated with reduced ITIH4 expression in the postmortem dorsolateral prefrontal cortex of controls [33].

SNPs with nominal association implicated several other plausible susceptibility genes for BD and SCZ (Table 1). These include *SATB2*, which is a highly conserved chromatin remodeling gene [34]. A previous animal study demonstrated that *SATB2* was an essential regulator of axonal connectivity in the developing neocortex [35]. In addition, mutations spanning *SATB2* have been reported in patients with neurodevelopmental disorders, including autism [36, 37].

The present SCZ-BD associated SNPs implicated three promising candidate genes for shared BD-SCZ etiology, i.e., *CACNB2*, *GRM3*, and *GRIN2A*. The gene *CACNB2* encodes an L-type voltage-gated calcium channel subunit, and is a reported genome-wide significant risk gene for several psychiatric disorders, including SCZ and BD [17].

The gene *GRM3* encodes a metabotropic glutamate receptor. *GRM3* is expressed predominantly in astrocytes, and has been investigated by previous authors as a potential therapeutic target in SCZ [14]. A further SCZ-BD SNP was located near *GRIN2A*, which encodes an NMDA receptor subunit involved in glutamatergic neurotransmission and synaptic plasticity [14]. Interestingly, rare mutations in *GRIN2A* have been reported in patients with SCZ [38].

The present pathway analysis implicated calcium- and glutamate signaling, and neuropathic pain signaling in dorsal horn neurons. These findings are consistent with previous pathway analyses of BD and SCZ [5, 25–27]. These results thus provide further evidence that neurotransmitter signaling and synaptic processes are involved in the development of BD and SCZ.

Our enrichment analysis identified a total of 25 enriched gene-sets, which showed partial overlap in terms of the underlying genes. One of the major characteristics of the GO database is its hierarchical structure. This structure involves the use of broad ‘parent’ terms, which can be divided into more distinctive ‘child’ terms [39]. After taking these relations into account, we categorized our findings from the GO database into five different parent gene-set families: channel activity, lipase activity, mitotic cell cycle, calmodulin binding, and glutamate receptor signaling (S3 Table).

The results generated by IPA and INRICH were broadly consistent, despite the fact that the underlying databases were different. In some cases, pathways were implicated by the same genes, e.g., glutamate signaling was implicated by *GRIN2A* and *GRM3* in both IPA and INRICH. In other cases, pathways were implicated by differing genes, e.g., calcium channel activity/calcium signaling was implicated by *NFATC3* and *GRIN2A* in IPA, and by *CACNB2* and *CACNA1C* in INRICH (S2 and S3 Tables). This provides further support for the involvement of these pathways in the development of BD and SCZ.

The most strongly enriched pathway according to IPA was synaptic long term potentiation (Fig 2). This pathway has been implicated in learning and memory mechanisms [40]. Interestingly, several previous studies have provided evidence for the involvement of impaired long term potentiation in the pathophysiology of SCZ [41, 42]. In the present study, this pathway result was driven by the genes *GRIN2A*, *GRM3*, and *CACNA1C*. The products of all three genes are located in the postsynaptic membrane (Fig 2), which may suggest that dysfunction at the postsynaptic level is an early step in the development of BD and SCZ [43].

The identified pathways support specific hypotheses regarding the shared neurobiology of BD and SCZ. Notably, our results provide further evidence that glutamate signaling might be involved in the development of both SCZ and BD [44]. This would be consistent with the observation from routine clinical practice that SCZ drugs which target glutamate signaling are also effective in BD patients with psychosis or mania [44].

A limitation of the present study was the substantial sample overlap between our BD GWAS [12] and the SCZ GWAS of the PGC [14], since this creates an inflation of effect. To address this, the correlation of z-scores between the two studies was calculated. Based on this information, the initial z-scores were then transformed to correct for sample overlap. To estimate the correlation of test statistics, the publically available summary statistics of the PGC SCZ GWAS were used, which comprise the results of the discovery phase (35,476 patients, 46,839 controls). As the effect of shared samples might be stronger in the discovery sample than in the complete meta-analysis, we may have overestimated the correlation of test statistics between the two GWAS. Therefore our correction for sample overlap may have been too conservative. However, since the inflation effect introduced by shared samples might be different for independent SNPs compared to the average correlation of test statistics, we assume that our conservative approach was appropriate in terms of reducing false positive results. In future cross-disorder studies, shared samples should be identified and removed from one study on the basis of individual genotype data. This was not possible in the present study, as the analyses were based on summary statistics.

The present data provide further insights into shared risk loci and disease-associated pathways for BD and SCZ.

However, further research is required to determine precisely how the genetic risk variants correlate with particular diagnoses or clinical symptoms. For example, in a previous study, we showed that common variation at the *NCAN* locus was associated with both BD [8] and SCZ [45]. Genetic variation at the *NCAN* locus thus represents a cross-diagnosis contributory factor, which may relate to a specific mania symptom-complex [46]. Therefore, future studies are warranted to determine the specific BD and SCZ phenotypic dimensions to which the present variants contribute. Such findings may suggest new research directions for the treatment and prevention of BD and SCZ.

## Supporting information

S1 Table Overview of the 107 investigated schizophrenia-associated SNPs and respective test statistics. Single nucleotide polymorphisms (SNPs) are shown according to their p-values in our bipolar disorder (BD) GWAS [12] following correction for sample overlap. Chromosomal positions refer to genome build GRCh37 (hg19). An imputation accuracy metric of 1 indicates that the respective SNP was not imputed using ImpG-Summary. Abbreviations: Chr, chromosome; A1, the allele to which the z-score is predicted; A2, other allele; Z/P BD Meta, z-score/p-value in our BD GWAS [12] after correction for sample overlap; Pcorr BD Meta, p-value in our BD GWAS [12] after correction for sample overlap and Bonferroni correction for multiple testing; Z/P PGC SCZ (discovery), derived z-score/p-value in the PGC schizophrenia GWAS (discovery phase) [14].
(XLSX)

S2 Table Results of the Ingenuity Pathway Analysis. Enrichment p-values for all nine nominally associated pathways containing two and more genes are shown both prior to and after Benjamini Hochberg (B-H) correction for multiple testing. Abbreviation: No. Genes in Pathway, total number of genes in each pathway.
(DOCX)

S3 Table Results of the INRICH pathway analysis. Empirical gene set p-values for all 16 nominally associated pathways containing two and more genes are shown. The p-values were corrected for multiple testing using bootstrapping-based re-sampling (corrected p-value). Abbreviations: GO, Gene Ontology; No. Genes in Pathway, total number of genes in each pathway.
(DOCX)
