# Supplementary material for: Identification of shared risk loci and pathways for bipolar disorder and schizophrenia
Source: PLoS One. 2017 Feb 6;12(2):e0171595. doi: 10.1371/journal.pone.0171595 (PMC5293228; doi:10.1371/journal.pone.0171595)
Supplement: S2 Table — Enrichment p-values for all nine nominally associated pathways containing two and more genes are shown both prior to and after Benjamini Hochberg (B-H) correction for multiple testing. Abbreviation: No. Genes in Pathway, total number of genes in each pathway. (DOCX) [file pone.0171595.s002.docx]

**S2 Table. Results of the Ingenuity Pathway Analysis**

| **Ingenuity Canonical Pathways** | **p-value** | **B-H p-value** | **Significant genes** | **No. Genes in Pathway** |
| --- | --- | --- | --- | --- |
| Synaptic Long Term Potentiation | 5.25x10^-5^ | 0.003 | *GRIN2A,CACNA1C,GRM3* | 119 |
| Glutamate Receptor Signaling | 0.001 | 0.019 | *GRIN2A,GRM3* | 57 |
| Amyotrophic Lateral Sclerosis Signaling | 0.002 | 0.030 | *GRIN2A,CACNA1C* | 98 |
| Neuropathic Pain Signaling in Dorsal Horn Neurons | 0.002 | 0.030 | *GRIN2A,GRM3* | 100 |
| Dopamine-DARPP32 Feedback in cAMP Signaling | 0.004 | 0.046 | *GRIN2A,CACNA1C* | 161 |
| CREB Signaling in Neurons | 0.005 | 0.046 | *GRIN2A,GRM3* | 171 |
| Ephrin Receptor Signaling | 0.005 | 0.046 | *GRIN2A,PAK6* | 174 |
| Calcium Signaling | 0.005 | 0.046 | *NFATC3,GRIN2A* | 178 |
| Axonal Guidance Signaling | 0.030 | 0.120 | *PAK6,NFATC3* | 434 |
