# Supplementary material for: Identification of shared risk loci and pathways for bipolar disorder and schizophrenia
Source: PLoS One. 2017 Feb 6;12(2):e0171595. doi: 10.1371/journal.pone.0171595 (PMC5293228; doi:10.1371/journal.pone.0171595)
Supplement: S3 Table — Empirical gene set p-values for all 16 nominally associated pathways containing two and more genes are shown. The p-values were corrected for multiple testing using bootstrapping-based re-sampling (corrected p-value). Abbreviations: GO, Gene Ontology; No. Genes in Pathway, total number of genes in each pathway. (DOCX) [file pone.0171595.s003.docx]

**S3 Table. Results of the INRICH pathway analysis**

| **GO gene sets** | **p-value** | **Corrected**  **p-value** | **Parent term** | **Significant genes** | **No. Genes in Pathway** |
| --- | --- | --- | --- | --- | --- |
| Voltage-gated calcium channel complex | 0.001 | 0.177 | Channel activity | *CACNB2,CACNA1C* | 14 |
| Voltage-gated calcium channel activity | 0.001 | 0.177 | Channel activity | *CACNB2,CACNA1C* | 17 |
| Calmodulin binding | 0.003 | 0.427 | Calmodulin binding | *NRGN,CACNA1C* | 25 |
| Calcium channel activity | 0.004 | 0.478 | Channel activity | *CACNB2,CACNA1C* | 31 |
| Voltage-gated cation channel activity | 0.008 | 0.623 | Channel activity | *CACNB2,CACNA1C* | 64 |
| Voltage-gated channel activity | 0.009 | 0.662 | Channel activity | *CACNB2,CACNA1C* | 70 |
| Phospholipase activity | 0.020 | 0.818 | Lipase activity | *PLCB2,PLA2G15* | 42 |
| Glutamate receptor activity | 0.021 | 0.829 | Glutamate receptor signaling | *GRM3,GRIN2A* | 20 |
| Lipase activity | 0.022 | 0.830 | Lipase activity | *PLCB2,PLA2G15* | 50 |
| Gated channel activity | 0.022 | 0.834 | Channel activity | *CACNB2,CACNA1C* | 116 |
| Cation channel activity | 0.024 | 0.850 | Channel activity | *CACNB2,CACNA1C* | 116 |
| M Phase | 0.029 | 0.882 | Mitotic cell cycle | *BOLL,PBRM1,CDC25C* | 112 |
| Ion channel activity | 0.032 | 0.895 | Channel activity | *CACNB2,CACNA1C* | 143 |
| Substrate specific channel activity | 0.034 | 0.903 | Channel activity | *CACNB2,CACNA1C* | 150 |
| Mitosis | 0.043 | 0.929 | Mitotic cell cycle | *PBRM1,CDC25C* | 81 |
| M Phase of mitotic cell cycle | 0.044 | 0.930 | Mitotic cell cycle | *PBRM1,CDC25C* | 84 |
